# Influence of per-O-sulfation upon the conformational behaviour of common furanosides

**DOI:** 10.3762/bjoc.15.63

**Published:** 2019-03-15

**Authors:** Alexey G Gerbst, Vadim B Krylov, Dmitry A Argunov, Maksim I Petruk, Arsenii S Solovev, Andrey S Dmitrenok, Nikolay E Nifantiev

**Affiliations:** 1 Laboratory of Glycoconjugate Chemistry, N.D. Zelinsky Institute of Organic Chemistry, Russian Academy of Sciences, Leninsky prospect 47, 119991 Moscow, Russia; 2 M.V. Lomonosov Moscow State University, Moscow, 119991, Russia

**Keywords:** ab initio calculations, conformational analysis, furanosides, NMR, sulfation

## Abstract

The studies on the recently discovered pyranoside-*into*-furanoside rearrangement have led us to conformational investigations of furanosides upon their total sulfation. Experimental NMR data showed that in some cases drastic changes of the ring conformation occurred while sometimes only the conformation of the exocyclic C4–C5 linkage changed. Herein we describe a combined quantum chemical and NMR conformational investigation of three common monosaccharide furanosides as their propyl glycosides: α-mannose, β-glucose and β-galactose. Full exploration of the furanoside ring by means of ab initio calculations was performed and coupling constants were calculated for each of the low-energy conformers. The results demonstrated preferred *trans*-orientation of H4–H5 protons in the non-sulfated molecules which changed to *gauche*-orientation upon sulfation. The effect is less pronounced in the galactosides. For all the studied structures changes in the conformational distribution were revealed by quantum mechanical calculations, that explained the observed changes in intraring coupling constants occurring upon introduction of sulfates.

## Introduction

Changes in the conformations of monosaccharides expectedly accompany their modification with different functional groups. Thus, spatial repulsion of silyl groups results in inversion or distortion of the pyranoside ring [1–2], which strongly modifies the chemical behaviour of the pyranoside substrate by influencing the spatial environment of the reaction center and changing stereoelectronic effects [3–5]. A rather complex case is observed for sulfate groups; in addition to van der Waals interactions their negative charges contribute to the electrostatic forces. The per-O-sulfation results in drastic conformational changes of pyranosides: β-glucopyranosides, β-xylopyranosides and β-glucuronides [6–9].

The furanosides are generally more conformationally flexible than pyranosides [10–11] and thus the effects of substitution in them are more complex. The conformational effects underlay the striking stereoselectivity in the glycosylation reaction by furanosyl donors [12]. Conformational analysis of furanosides includes both conformation of the furanoside ring and conformation of the side chain at C(4). The conformation of the furanoside ring can be one of ten envelope (E) or ten twist (T) forms and it is convenient to describe it using the *pseudo*-rotation diagram [13]. The conformation of the exocyclic chain (i.e., rotation of C4−C5 and C5−C6 bonds) is described by two torsion angles [10].

The knowledge of conformational changes occurring in sulfated furanosides may be important for better understanding of the driving force of the pyranoside-*into*-furanoside rearrangement [14–16], which is widely used for preparative synthesis of different oligosaccharides, including fragments of a galactomannan from *Aspergillus* fumigatus [17–19], diheteroglycan from *Enterococcus faecalis* [20], galactan I from *Klebsiella pneumoniae* [21] and fucoidan from brown seaweed *Chordaria flagelliformis* [22].

In this communication the conformational analysis of three common per-O-sulfated furanosides is reported.

## Results and Discussion

### Synthesis of per-O-sulfated furanosides

**Scheme 1:**
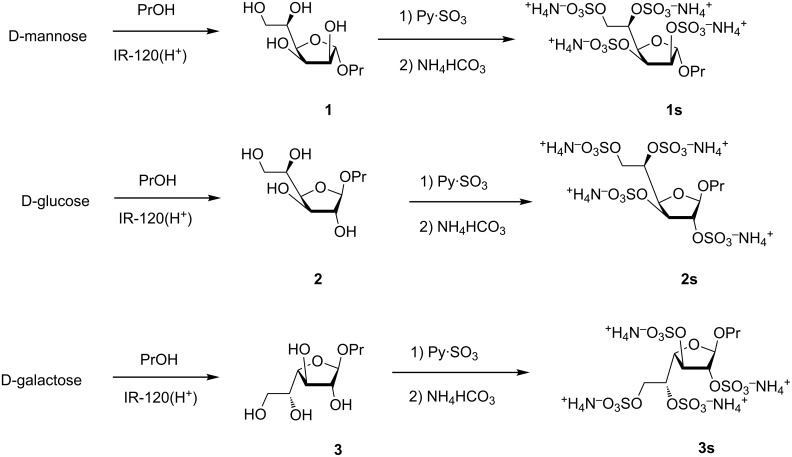
Studied monosaccharides **1**–**3** and **1s**–**3s** and their preparation.

Propyl α-D-mannofuranoside (**1**) was prepared from D-mannose and *n*-propanol via Fischer reaction using ion-exchange resin IR-120(H^+^) as acidic catalyst. The reaction was performed under kinetic control and was stopped at low conversion of the starting mannose to avoid formation of the pyranoside product [23]. The desired furanoside **1** was isolated from the reaction mixture by column chromatography with a yield of 12%. Parent propyl β-D-glucofuranoside (**2**) and propyl β-D-galactofuranoside (**3**) were prepared using analogous reactions (Scheme 1). The use of the *n*-propyl group as an aglycon allowed for efficient purification of the desired glycosides utilizing C18 reversed-phase chromatography. Galactofuranoside **3** was previously synthesized using pyranoside-*into*-furanoside rearrangement [14].

The per-O-sulfation [24–25] of parent furanosides **1**–**3** was performed by their treatment with an excess of Py·SO_3_ complex in DMF. After the reaction was finished, the reaction mixture was neutralized with aqueous NH_4_HCO_3_, concentrated in vacuo, dried and used for further NMR analysis without additional purification (Scheme 1).

### NMR data of furanosides **1**–**3** and **1s**–**3s**

^1^H and ^13^C NMR spectra of parent monosaccharides **1**–**3** and their per-O-sulfated derivatives **1s**–**3s** were recorded in D_2_O. The signal assignment was performed using 2D COSY and HSQC. *J* coupling constants were measured directly from 1D ^1^H NMR spectra. In case of overlapping signals *J* coupling constants were extracted from 2nd order spectra simulations using Bruker TopSpin software (DAISY). The obtained results (see Tables 1–3) showed good coincidence with previously published data for related monosaccharides [15,26–27].

**Table 1:** ^1^H NMR chemical shifts of non-sulfated (**1**–**3**^a^) and per-O-sulfated (**1s**–**3s**^b^) propyl furanosides.

compound	^1^H (δ, ppm)						

	H(1)	H(2)	H(3)	H(4)	H(5)	H(6a)	H(6b)

**1**	4.96	4.07	4.24	3.98	3.84	3.72	3.54
**1s**	5.33	4.77	5.29	4.79	4.89	4.47	4.17
**2**	4.89	4.05	4.15	4.06	3.89	3.76	3.59
**2s**	5.29	5.05	5.03	4.74	4.88	4.55	4.24
**3**	4.92	3.97	4.00	3.88	3.74	3.63	3.58
**3s**	5.36	4.83	5.05	4.44	4.90	4.35	4.25

^a 1^H chemical shifts for the propyl aglycon in non-sulfated compounds: H(1а) = 3.63; H(1b) = 3.47; H(2) = 1.51; H(3) = 0.82 ppm; ^b 1^H chemical shifts for the propyl aglycon in per-O-sulfated compounds: H(1а) = 3.69; H(1b) = 3.59; H(2) = 1.61; H(3) = 0.91 ppm.

**Table 2:** ^13^C NMR chemical shifts of non-sulfated (**1**–**3**^a^) and per-O-sulfated (**1s**–**3s**^b^) propyl furanosides.

compound	^13^C (δ, ppm)					

	C(1)	C(2)	C(3)	C(4)	C(5)	C(6)

**1**	107.4	76.7	71.1	79.1	69.0	63.0
**1s**	104.0	78.3	75.0	77.1	76.2	66.9
**2**	108.0	79.6	74.7	80.9	69.5	63.4
**2s**	106.9	83.0	78.9	80.8	75.8	67.8
**3**	107.0	80.9	76.4	82.3	70.8	62.8
**3s**	105.5	84.9	81.4	81.4	74.6	66.3

^a 13^C chemical shifts for the propyl aglycon in non-sulfated compounds: C(1) = 71.4; C(2) = 22.2; C(3) = 9.7 ppm; ^b 13^C chemical shifts for the propyl aglycon in per-O-sulfated compounds: C(1) = 70.4; C(2) = 22.1; C(3) = 9.9 ppm.

**Table 3:** ^1^H–^1^H NMR *J* coupling constants of non-sulfated (**1**–**3**) and per-O-sulfated (**1s**–**3s**) propyl furanosides.

compound	*J* constants (Hz)						

	*J*_1,2_	*J*_2,3_	*J*_3,4_	*J*_4,5_	*J*_5,6a_	*J*_5,6b_	*J*_6a,6b_

**1**	4.6	4.6	2.9	8.8	2.8	6.2	−12.1
**1s**	1.2	5.6	7.0	2.8	2.4	7.9	−11.4
**2**	<1	1.2	4.5	9.0	2.8	6.1	−12.0
**2s**	<1	<1 (≈0.7)	4.8	4.9	2.3	5.8	−11.2
**3**	2.4	4.4	6.8	4.0	4.3	7.5	−11.7
**3s**	<1	<1	4.6	2.4	5.3	7.5	−10.4

As can be seen from Table 3, *J* coupling constants significantly differ for non-sulfated and per-*O*-sulfated furanosides which allows for a conclusion that their conformations are changed after the sulfation. To rationalize these changes, we undertook theoretical conformational analysis of monosaccharides **1**–**3** and **1s**–**3s**.

### Conformational analysis of the furanoside ring

For the theoretical exploration of the conformational properties of the furanoside rings several starting structures were generated for each compound. They represented all possible furanoside *pseudo*-rotamers. Additionally, for each of these structures the torsional angle H4–C4–C5–H5 defining the conformation of the exocyclic C4–C5 bond was set either to +60°, −60° or 180°. The only exception was the sulfated mannoside **1s**, where ^3^*J*_H4-H5_ was very small suggesting low contribution of the 180° conformer. The starting conformation of the propyl aglycon was always chosen as follows: O4–C1–O1–CH_2_ torsion was set to +60° or −60° depending on α- or β-configuration of the sugar in accordance with the *exo*-anomeric effect. Other starting torsions in the propyl group had *trans*-orientation. Geometry optimizations of all the thus obtained structures were carried out at ab initio HF/6-311++G** level using the COSMO continual solvation model with parameters for water. For complete computational details see the Experimental part.

For all the three studied monosaccharides, both in non-sulfated (**1**–**3**) and sulfated (**1s**–**3s**) forms, the geometry optimizations tended to produce one or two low-energy conformers which differed from each other by less than 2 kcal/mol. The other conformations found had considerably higher energies. For non-sulfated structures **1–3** sometimes high-energy conformations were obtained with the same puckering state of the furanoside ring (see Tables in Supporting Information File 1). Examination of these conformations revealed that these deviations were due to unfavorable orientation of the 2-OH and 3-OH hydroxy groups. In these cases re-optimization of the high-energy conformer was performed to ensure that energy would converge to the minimum.

**Figure 1:**
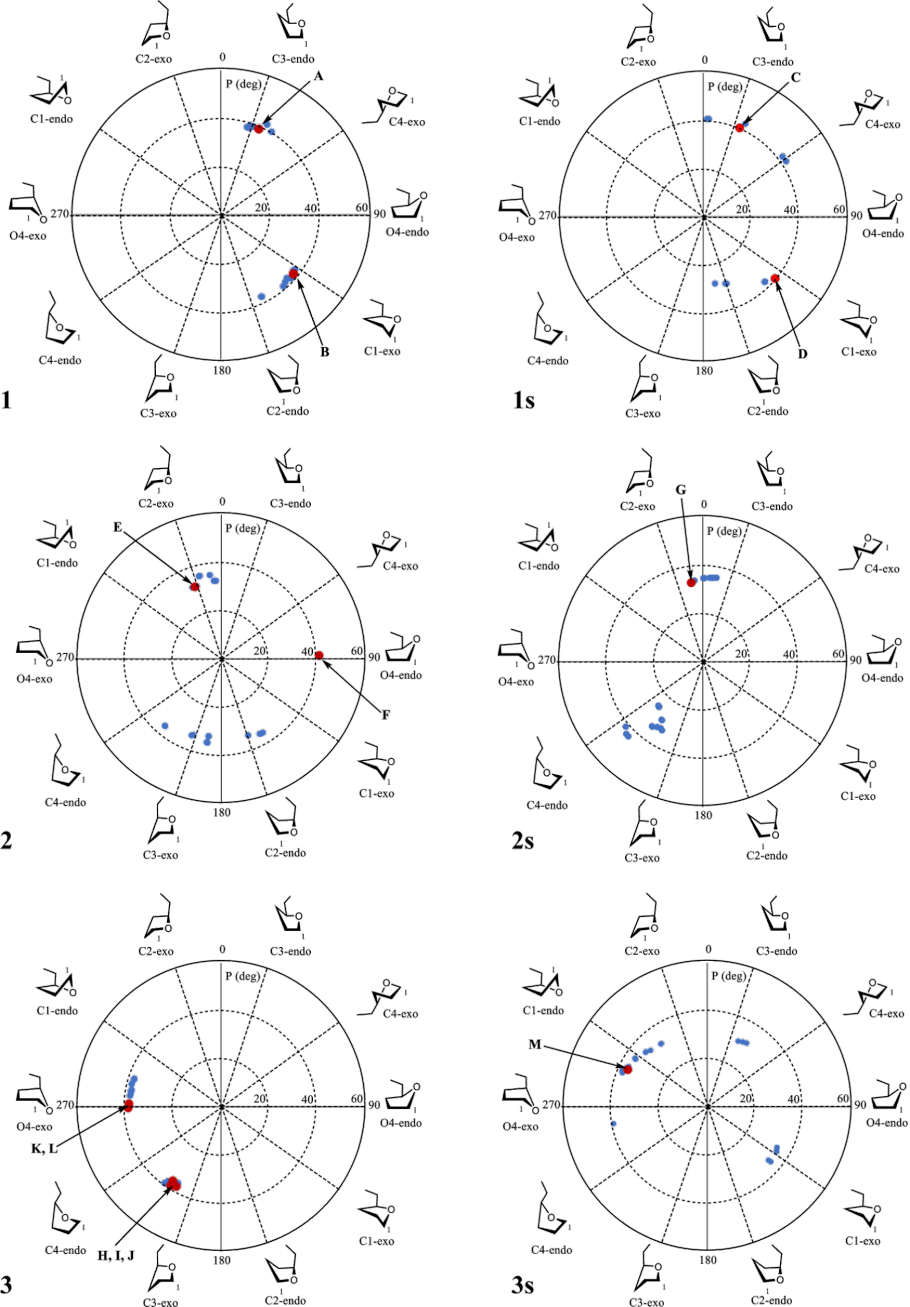
The *pseudo*-rotation wheels showing different optimized structures of furanosides **1**–**3** and **1s**–**3s**. The lower energy conformations (denoted as in Table 4) are colored in red.

**Figure 2:**
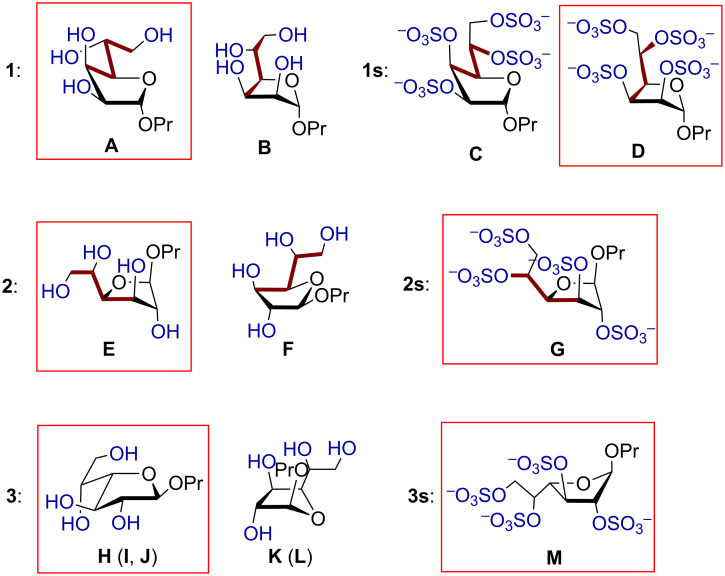
Schematic views of low energy conformers **A**–**M**. The minimal energy conformers are embedded in red frames. The substituents at C4–C5 bond located in *trans*-position are shown in brown. The conformers H–J and K, L only differ in the H4–C4–C5–H5 dihedral angle (see Table 4).

Table 4 shows the descriptions of all the low-energy conformations obtained. The whole list of the resulting conformers can be found in Supporting Information File 1. All the obtained conformers are plotted on the *pseudo*-rotation wheel diagrams, where low-energy conformers are shown in red dots (Figure 1). Schematic views of the obtained low-energy conformers are shown in Figure 2.

**Table 4:** Low-energy conformers obtained after ab initio geometry optimizations of compounds **1**–**3** and **1s**–**3s**.

compound		main low-energy conformers	value of H4–C4–C5–H5 dihedral	relative energy, kcal/mol^a^	conformer notation	P	ν

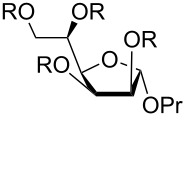	R = H	C3-*endo*	+179°	0.0	**A**	23	39
	R = H	C1-*exo*	+178°	0.7	**B**	130	38
	R = SO_3_^−^	C3-*endo*	+84°	1.2	**C**	22	40
	R = SO_3_^−^	C1-*exo*	+70°	0.0	**D**	131	39
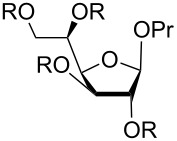	R = H	C2-*exo*	+174°	0.0	**E**	−20	32
	R = H	O4-*endo*	+173°	0.2	**F**	88	41
	R = SO_3_^−^	C2-*exo*	+84°	0.0^b^	**G**	−8	33
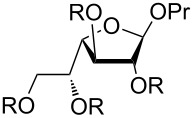	R = H	C3-*exo*	−57°	0.0	**H**	−148	39
	R = H	C3-*exo*	+53°	1.0	**I**	−151	38
	R = H	C3-*exo*	+173°	1.5	**J**	−147	37
	R = H	O4-*exo*	−59°	1.2	**K**	−91	38
	R = H	O4-*exo*	+52°	1.8	**L**	−88	39
	R = SO_3_^−^	C1-*endo*	−63°	0.0^b^	**M**	−65	37

^a^Values relative to the lowest energy conformer for each structure are given. ^b^The conformer with minimal relative energy is presented. The other conformers can be found in Supporting Information.

From Table 4 it can be seen that in case of the mannoside **1** and glucoside **2**, in the absence of sulfates, the preferable conformation of the C4–C5 bond is characterized with *trans*-orientation of H4 and H5 protons. However, for their sulfated derivatives **1s**, **2s** the *gauche*-rotamer was dominant. This observation is obviously connected with the introduction of sulfate at O5. Thus, in the non-sulfated form the most bulky group at C(5) is CH_2_OH which prefers to adopt the *trans*-orientation to the C3 atom of the ring. However, in sulfated derivatives the SO_3_^−^ group at O5, due to its size, tends to have *trans*-orientation to C(3) (see Figure 2). In the case of galactose **3**, however, the situation is more complex. This saccharide in its furanoside form has increased conformational flexibility around the C4–C5 bond, because for its lowest energy conformer (C3-*exo*) all three rotamers (**H**, **I**, **J**) do not have great energy difference between each other. For its other ring conformer, O4-*exo*, two C4–C5 rotamers (**K**, **L**) are possible according to the calculation results.

For all the examined structures changes in the ring conformation upon the introduction of sulfates are observed. Particularly, in the mannoside prevalence of the conformers changes: while in the free form of **1** the calculations predict it to exist preferably in C3-*endo* conformation (Table 4, conformer **A**), in the sulfated form the C1-*exo* conformer becomes dominant (conformer **D**). In the glucoside, the conformation of the furanoside ring in the conformer with minimal energy stays approximately the same (C2-*exo* for both **E** and **G** conformers), however, O4-*endo* conformer (conformer **F**) which is present in non-sulfated form **2** disappears in the sulfated one (**2s**). In case of the galactosides the low energy C3-*exo* conformers (**H**, **I**, **J**) which should dominate in non-sulfated form **3** disappear after introduction of O-sulfates and conformational shift to C1-*endo* (conformer **M**) occurs.

Additionally, pseudorotational analysis of compounds **1**–**3** and **1s**–**3s** was performed using the MatLab program developed by P. M. S. Hendrickx and J. C. Martins [28]. The results are shown in Table 5. Comparison of the ring puckering parameters obtained from this analysis with those for the lowest energy conformers in Table 4 shows that they are of the same range and demonstrate the same tendencies as found from quantum mechanical calculations.

**Table 5:** Best-fit conformers obtained after pseudorotaional analysis of compounds **1**–**3** and **1s**–**3s**.

compound	best-fit conformer parameters (found by MatLab [28])		RMSD (Hz)

	P	ν	

**1**	20.4	45.3	0.61
**1s**	184.5	44.2	0.32
**2**	6.2	33.3	0.36
**2s**	29.9	35.0	0.01
**3**	−155.2	20.4	0.21
**3s**	−64.1	35.5	0.05

All the mentioned changes certainly affect the values of the intraring ^1^H–^1^H coupling constants. To study this influence in detail, DFT/B3LYP/pcJ-1 calculation of the constants for low-energy conformers (**A**–**M**) denoted in Table 4 was performed (Table 6). The first thing to note is that for non-sulfated α-propyl mannofuranoside **1** in the lowest-energy conformer **A** all the computed intraring constants, and, to some extent, ^3^*J*_4,5_ reproduce the experimental values (Table 6, entries 1 and 2). According to the calculations the drastic decrease of the experimentally measured H1–H2 coupling constant in the mannofuranoside upon sulfation arises from the change of the conformational preference towards C1-*exo* (conformer **D**) in the sulfated saccharide.

The changes in conformation of side chain from *trans* into *gauche* rotamers (H4–C4–C5–H5 dihedral angle) upon the sulfation were clearly seen from the ^3^*J*_4,5_ coupling constants. Additionally to justify that the correct conformers for C4–C5 bond were obtained, two J-HMBC experiments were performed to measure the the H4–C6 ^3^*J*_C-H_ coupling whose value could distinguish between the three possible rotamers. Reasonable coincidence with the calculations was obtained: for the non-sulfated mannoside **1** the measured constant was 3 Hz (calcd. 3.6 Hz) and for the sulfated compound **1s** it was 5 Hz (calcd. 5.7 Hz).

The same calculations of coupling constants were performed for the glucosides and galactosides. In the case of the non-sulfated compounds **2** and **3** (Table 6) combination of the coupling constants calculated for the found conformers generally reproduced the experimental values of the intraring couplings. The only exception was conformer **F** of the glucoside **2** (Table 6, entry 9), whose ^3^*J*_H1-H2_ coupling is quite large while its relative energy is comparable (although still higher) than that of the main conformer **E** (Table 6, entry 8).

Good agreement between the theoretical and experimental data was obtained also in the case of the sulfated compounds **2s** and **3s** (Table 6, entries 10, 11, 18 and 19). However, it needs to be mentioned, that among other constants calculated for conformer **G** of **2s** (Table 6, entry 10), ^3^*J*_H4-H5_ deviates strongly from that measured experimentally (Table 6, entry 10). We attribute it to the fact that no counter-ions were considered in the calculations and thus the energies of the other rotamers around C4–C5 linkage could have been overestimated.

**Table 6:** Experimental ^1^H–^1^H coupling constants (Hz) and those calculated for different conformers (Hz) for furanosides **1**–**3** and **1s**–**3s**.

entry	compound	conformer notation	relative energy, kcal/mol	*J*_1,2_	*J*_2,3_	*J*_3,4_	*J*_4,5_

1	**1**	experimental	–	4.6	4.6	2.9	8.8
2		conformer **A**	0.0	4.9	4.4	2.6	9.6
3		conformer **B**	1.6	0.3	5.5	8.3	9.8
4	**1s**	experimental	–	1.2	5.6	7.0	2.8
5		conformer **D**	0.0	0.3	6.9	9.9	1.8
6		conformer **C**	1.2	5.6	5.0	2.7	0.5
7	**2**	experimental	–	<1	1.2	4.5	9.0
8		conformer **E**	0.0	0.1	0.5	5.4	10.0
9		conformer **F**	0.2	5.4	1.4	5.0	9.5
10	**2s**	experimental	–	<1	0.7	4.8	4.9
11		conformer **G**	0.0	0.1	0.8	5.9	0.5
12	**3**	experimental	–	2.4^a^	4.4^a^	6.8^a^	4.0^a^
13		conformer **H**	0.0	4.8	8.5	9.4	1.6
14		conformer **I**	1.0	5.2	8.1	9.5	6.3
15		conformer **J**	1.5	4.8	7.2	9.6	8.4
16		conformer **K**	1.2	0.3	1.4	6.9	6.4
17		conformer **L**	1.8	0.3	1.3	6.5	1.7
18	**3s**	experimental	–	<1	<1	4.6	2.4
19		conformer **M**	0.0	0.1	0.4	3.9	1.4

^a^The constant was obtained by second order spectrum simulation in Topspin DAISY.

**Figure 3:**
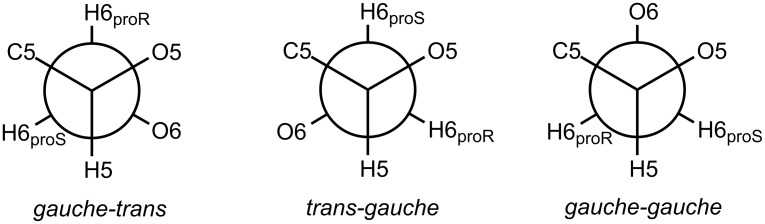
Denotation of conformers obtained during rotation around C5–C6 bond.

The rotation around the C5–C6 bond in furanosides obviously results in additional conformers. The investigation of conformational behavior of acyclic polyols was not the primary goal of this study, but to make sure that the rotation around C5–C6 is free and does not influence the conformation of the furanoside ring and thus the intraring couplings, the additional conformations were considered for mannosides **1** and **1s** (Table 7). The previously found conformers (**A** and **B** for the free mannoside **1** and **C** and **D** for its sulfated counter-part **1s**) had the C5–C6 bond in *gauche-trans* orientation (denotation of conformers see on Figure 3). The geometry optimizations showed that *gauche-gauche* conformers are also possible (Table 7, entries 3, 5, 9 and 11) and for the non-sulfated mannoside **1** *trans-gauche* rotamer was also found (Table 7, entry 6). It can be seen that, indeed, the rotation around this bond quite expectedly changes the values of the H5–H6 coupling constants, but additionally it significantly influences the vicinal H6_proR_-H6_proS_ coupling. The values of the intra-ring constants as well as *J*_4,5_ during the rotation change very slightly or do not change at all.

**Table 7:** Experimental ^1^H–^1^H coupling constants (Hz) for α-propyl mannofuranosides (**1**, **1s**) and those calculated for its different conformers (Hz).

entry	conformer notation and orientation of C5–C6 bond	relative energy, kcal/mol	*J*_1,2_	*J*_2,3_	*J*_3,4_	*J*_4,5_	*J*_5,6a_	*J*_5,6b_	*J*_6a,6b_^a^

1	experimental for **1**	–	4.6	4.6	2.9	8.8	2.8	6.2	12.1
2	conformer **A**, *gauche-trans*	0.0	4.9	4.4	2.6	9.6	2.5	10.3	−8.9
3	conformer **A**, *gauche-gauche*	1.3	4.8	4.5	2.7	9.8	2.7	1.4	−10.6
4	conformer **B**, *gauche-trans*	0.7	0.3	5.7	8.1	10.1	2.5	10.2	−9.4
5	conformer **B**, *gauche-gauche*	2.0	0.3	5.6	8.4	10.3	2.7	1.5	−11.2
6	conformer **B**, *trans-gauche*	1.6	0.3	5.5	8.3	9.8	10.1	3.1	−12.2
7	experimental for **1s**	–	1.2	5.6	7.0	2.8	2.4	7.9	11.4
8	conformer **D**, *gauche-trans*	0.0	0.3	6.9	9.9	1.8	1.7	10.2	−11.8
9	conformer **D**, *gauche-gauche*	3.6	0.3	7.0	10.0	1.9	5.8	0.5	−12.1
10	conformer **C**, *gauche-trans*	1.2	5.6	5.0	2.7	0.5	1.9	10.0	−11.2
11	conformer **C**, *gauche-gauche*	3.6	6.2	4.9	2.7	0.6	5.6	0.5	−11.5

^a^The sign of the constant was not determined experimentally.

## Conclusion

Conformational analysis of several common monosaccharides in the furanoside form was performed in order to study molecular geometry changes occurring upon total sulfation. It was found that these changes generally affected either the furanoside ring conformation or the conformation of C4–C5 side bond. Particularly, all the studied structures exhibited preference for *trans*-placement of H4 and H5 protons in the non-sulfated form which changed to *gauche*-orientation upon the introduction of sulfates. This tendency was less pronounced in the galactosides where in the free form all three C4–C5 rotamers were found to have rather low energies. The mannoside in the free form was theoretically predicted to exist preferably in C3-*endo* conformation while in the sulfated form C1-*exo* conformer became dominant. In the glucoside, O4-*endo* conformer disappeared in the sulfated form and in the galactoside conformational shift to C1-*endo* occurred.

## Experimental

### General procedures

Commercial chemicals were used without purification unless noted. All solvents for reactions were purchased as dry (DMF, *n*-propanol) solvents for chromatography (EtOAc, MeOH, H_2_O) were HPLC grade. Thin-layer chromatography (TLC) was carried out on aluminum sheets coated with silica gel 60 F_254_ (Merck). Analysis TLC plates were developed by treatment with a mixture of 15% H_3_PO_4_ and orcinol (1.8 g/L) in EtOH/H_2_O (95:5, v/v) followed by heating.

### NMR and computational studies

^1^H and ^13^C NMR spectra were recorded on Bruker AV-400 or Bruker Fourier 300HD spectrometers equipped with 5 mm pulsed-field-gradient (PFG) probes at temperatures denoted in the spectra in Supporting Information File 1. The resonance assignment in ^1^H and ^13^C NMR spectra was performed using 2D experiments COSY and HSQC. Chemical shifts are reported in ppm. NMR spectra were obtained using a standard pulse sequence from the Bruker software. In *J*-HMBC experiments the delay for the long-range couplings was optimized for 1.5 Hz. All spectra were transformed and analyzed with the Bruker Topspin 3.6 software.

Geometry optimization were performed using the ORCA 2.9.1 program [29–30]. RHF approximation with 6-311++G** basis set was employed [31]. Sulfates in the studied structures were treated as anions without presence counterions. COSMO [32] model was applied with built-in parameters for water. Geometry optimizations were performed until the RMS gradient reached a value less than 10^−4^. Coupling constants were computed using DFT/B3LYP/pcJ-1 [33] approximation and DALTON-2015 software [34]. Only Fermi-contact terms were evaluated.

### Synthesis of propyl α-D-mannofuranoside (**1**)

To a suspension of D-mannose (400 mg, 2.22 mmol) in *n*-propanol (4 mL) was added resin IR-120(H^+^) (475 mg). The mixture was heated to 80 °C and stirred for 4 h. Then the resin was filtered off. The residue was purified by column chromatography (EtOAc/MeOH 20:1) to afford compound **1** (59 mg, 12%) as a colorless syrup. *R*_f_ = 0.32 (EtOAc/MeOH 10:1).

### Synthesis of propyl β-D-glucofuranoside (**2**) and β-D-galactofuranoside (**3**)

The preparations of furanosides **2** and **3** were performed as described above for mannofuranoside **1**, however, the HPLC (C-18, eluted with MeOH/H_2_O) was additionally applied to separate α- and β-furanosides. The resulted yields of furanosides **2** and **3** were 10% and 24%, respectively.

### General protocol for per-O-sulfation

A solution of furanoside (0.02 mmol) and Py·SO_3_ (0.4 mmol) in DMF (500 μL) was stirred at 25 °C for 30 min. Then the reaction mixture was neutralized with an aqueous solution of NH_4_HCO_3_, concentrated in vacuo and then co-evaporated with H_2_O. The residue was dissolved in D_2_O (550 μL) and used for recording of NMR spectra.

## Supporting Information

File 1 Copies of ^1^H and ^13^C NMR spectra of compounds **1**–**3** and **1s**–**3s** and computational details for all found conformers.
