# Supplementary material for: Influence of per-O-sulfation upon the conformational behaviour of common furanosides
Source: Beilstein J Org Chem. 2019 Mar 15;15:685–94. doi: 10.3762/bjoc.15.63 (PMC6423562; doi:10.3762/bjoc.15.63)
Supplement: File 1 — Copies of 1H and 13C NMR spectra of compounds 1–3 and 1s–3s and computational details for all found conformers. [file Beilstein_J_Org_Chem-15-685-s001.pdf]

BEILSTEIN JOURNAL OF ORGANIC CHEMISTRY

Supporting Information

for

Influence of per-O-sulfation upon the conformational behaviour of common furanosides

Alexey G. Gerbst, Vadim B. Krylov, Dmitry A. Argunov, Maksim I. Petruk, Arsenii S. Solovev, Andrey S. Dmitrenok and Nikolay E. Nifantiev

Beilstein J. Org. Chem. **2019,** *15,* 685–694. doi:10.3762/bjoc.15.63

Copies of ^{1}H and ^{13}C NMR spectra of compounds 1–3 and 1s–3s and computational details for all found conformers

License and Terms: This is a supporting information file under the terms of the Creative Commons Attribution License (http://creativecommons.org/licenses/by/4.0). Please note that the reuse, redistribution and reproduction in particular requires that the authors and source are credited.
The license is subject to the *Beilstein Journal of Organic Chemistry* terms and conditions: (https://www.beilstein-journals.org/bjoc)

Propyl α-D-mannofuranoside (1)

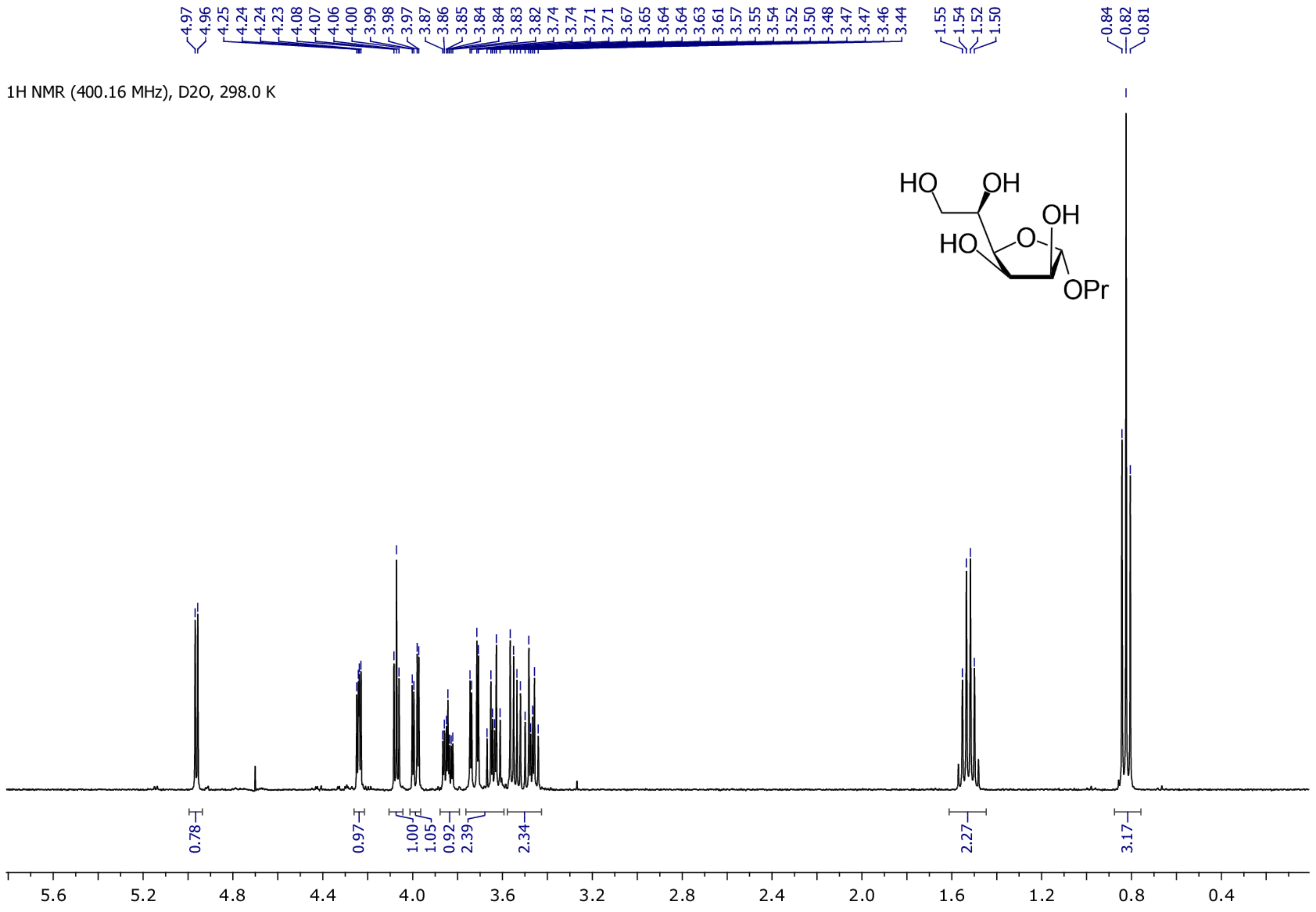

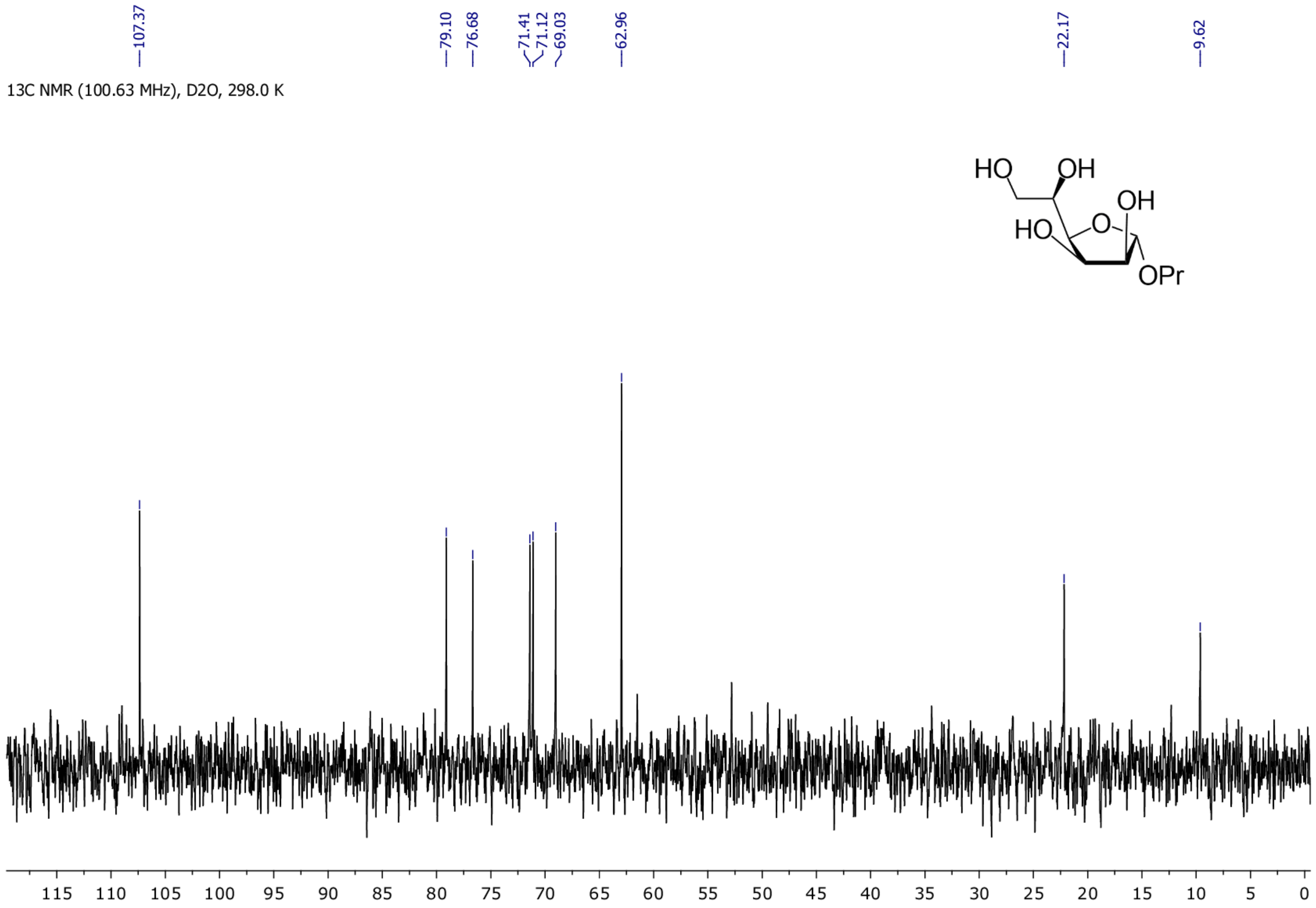

107.37
79.10
76.68
71.41
71.12
69.03
62.96
22.17
9.62
13C NMR (100.63 MHz), D2O, 298.0 K
HO
OH
OH
HO
O
OPr
115
110
105
100
95
90
85
80
75
70
65
60
55
50
45
40
35
30
25
20
15
10
5
0

Per-O-sulfated propyl α-D-mannofuranoside (1s)

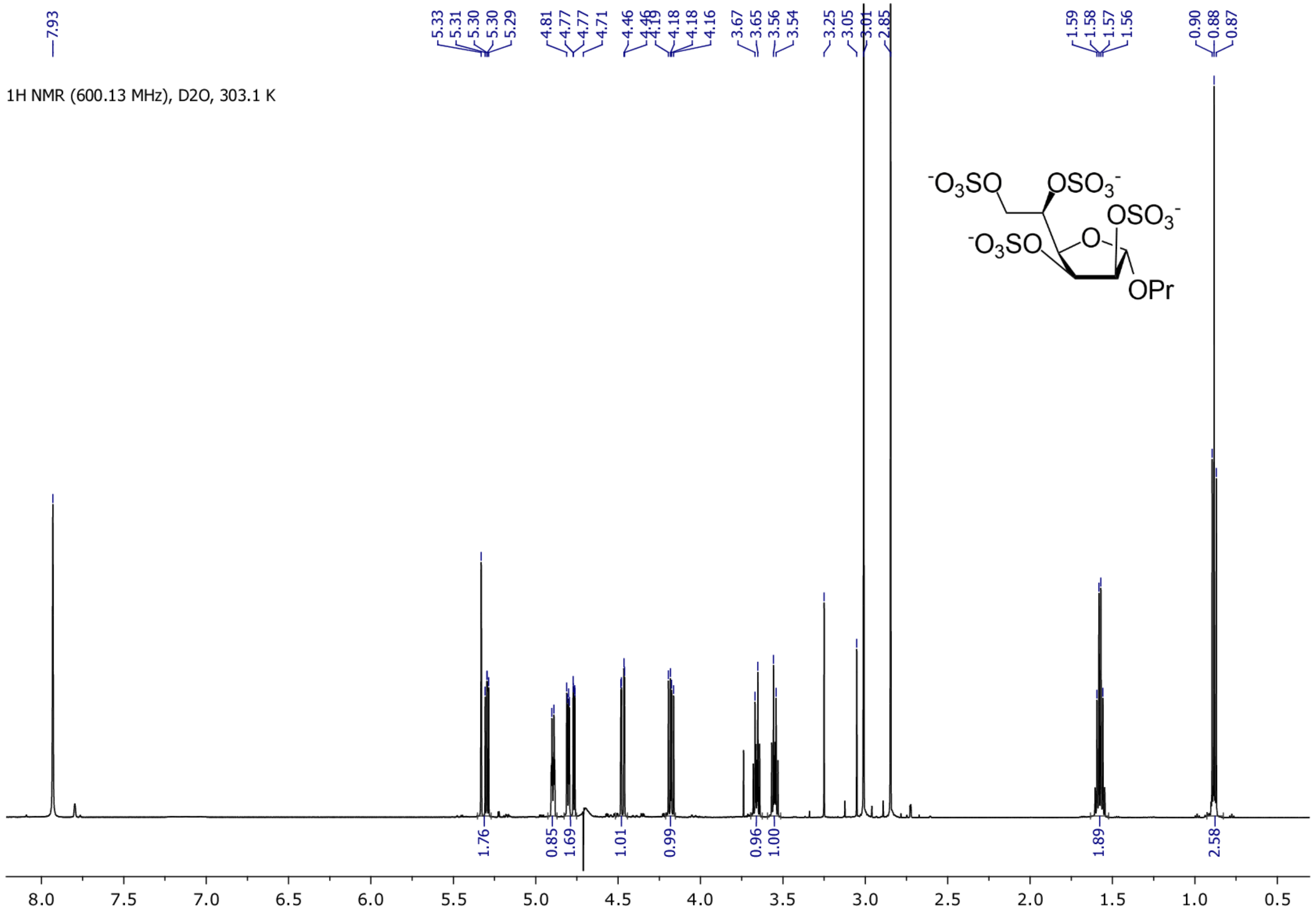

13C NMR (150.92 MHz), D2O, 303.2 K

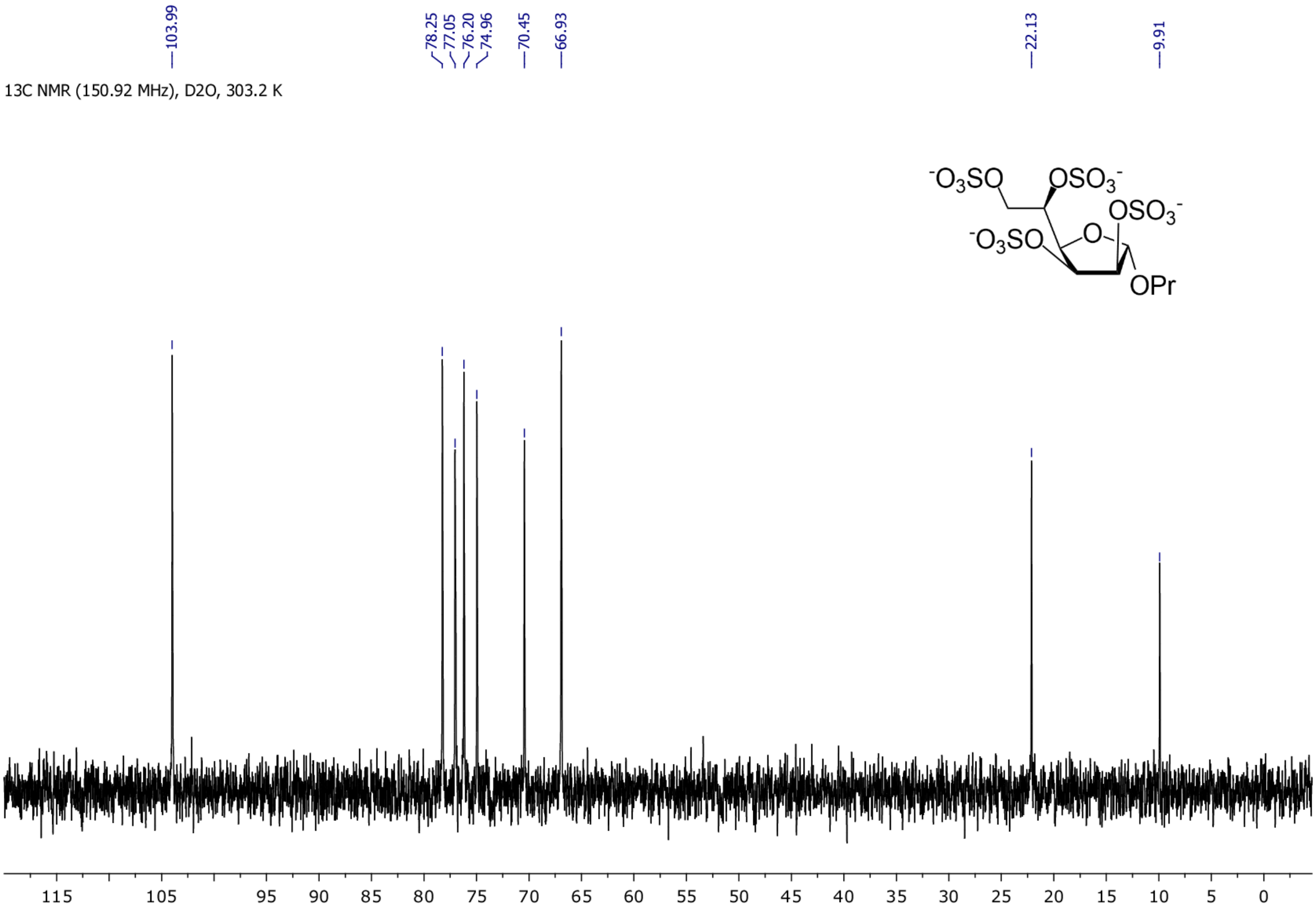

Propyl β-D-glucofuranoside (2)

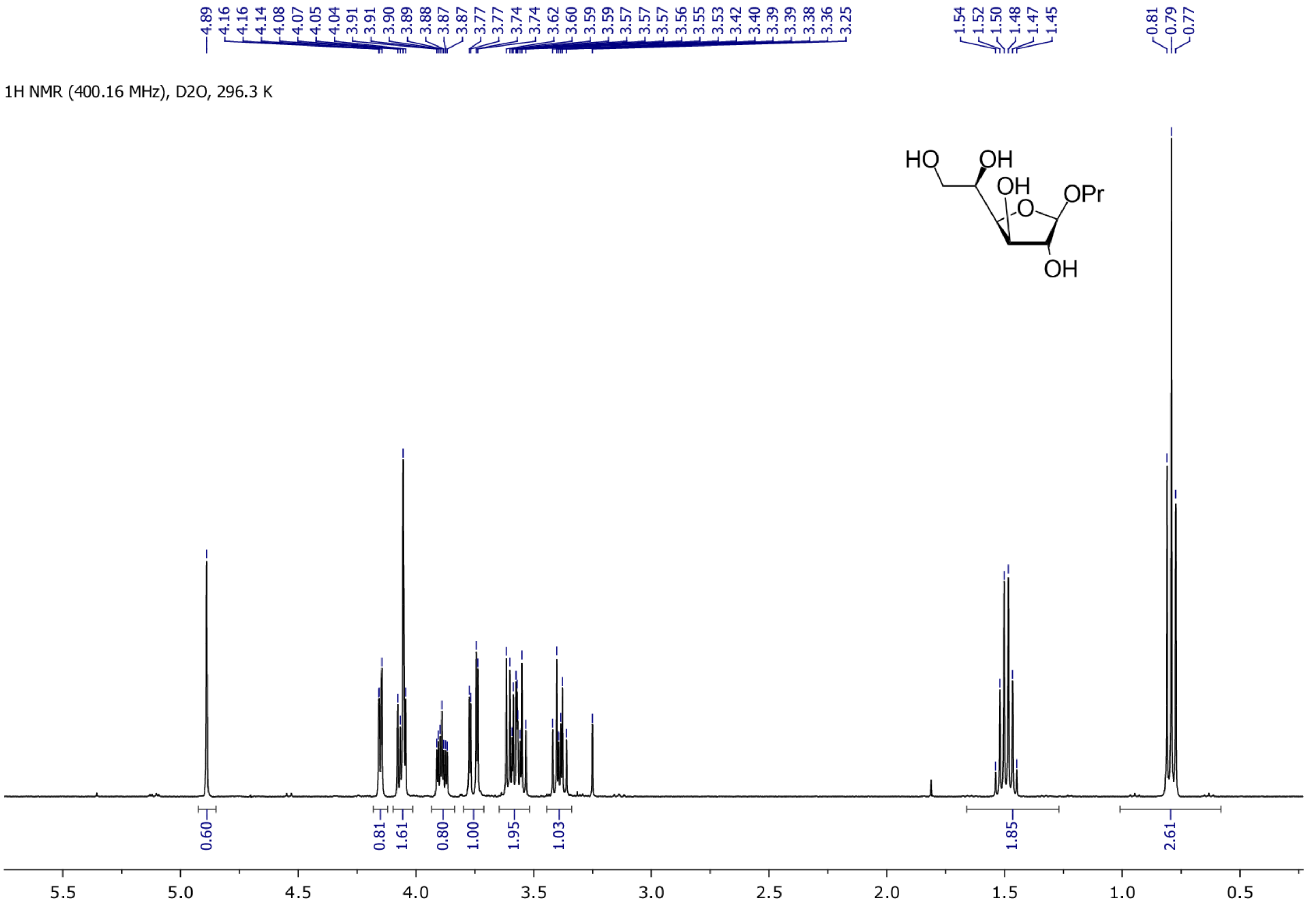

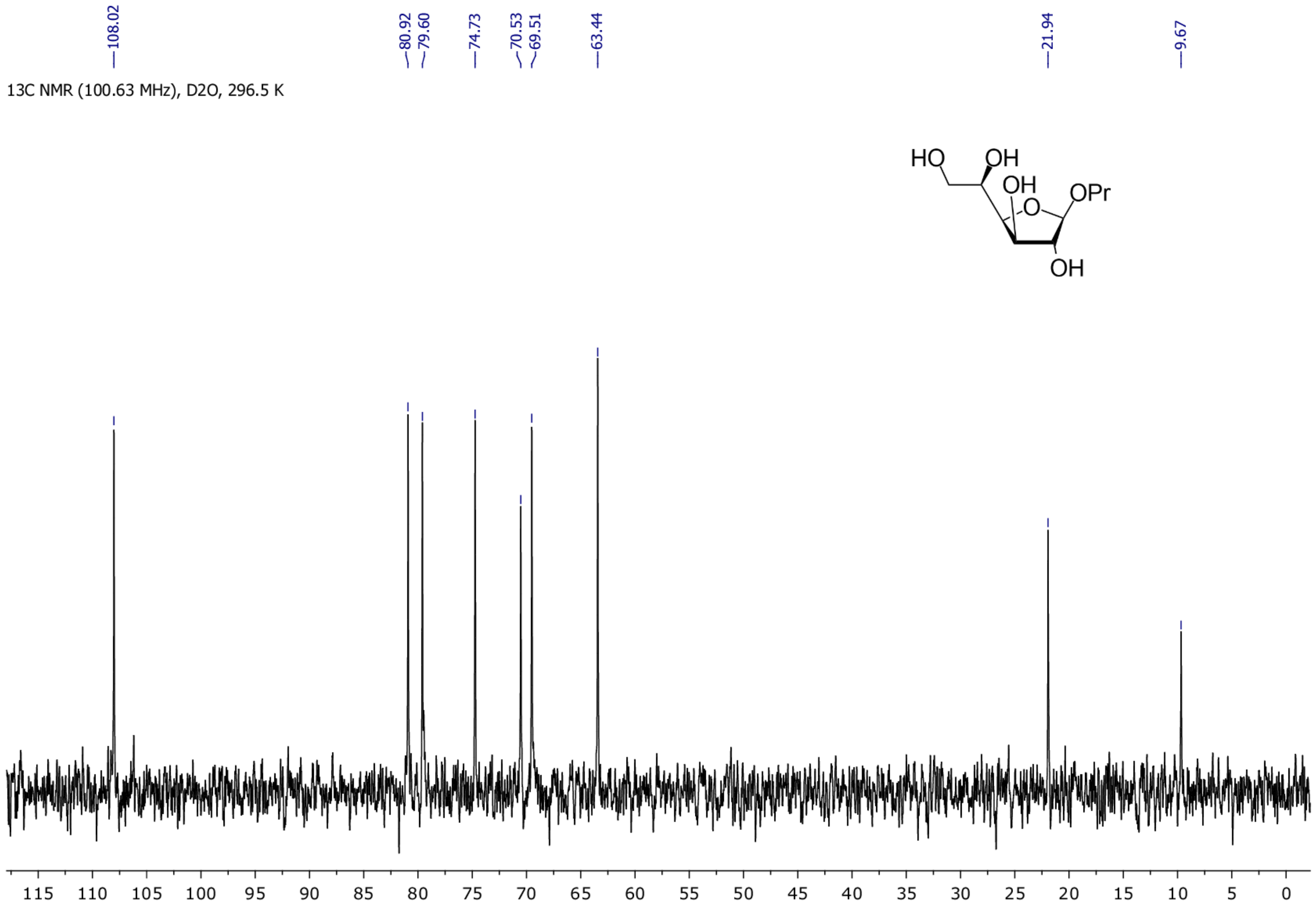
108.02
80.92
79.60
74.73
70.53
69.51
63.44
21.94
9.67
13C NMR (100.63 MHz), D2O, 296.5 K
HO
OH
OH
O
OPr
OH
115 110 105 100 95 90 85 80 75 70 65 60 55 50 45 40 35 30 25 20 15 10 5 0

Per-O-sulfated propyl β-D-glucofuranoside (2s)

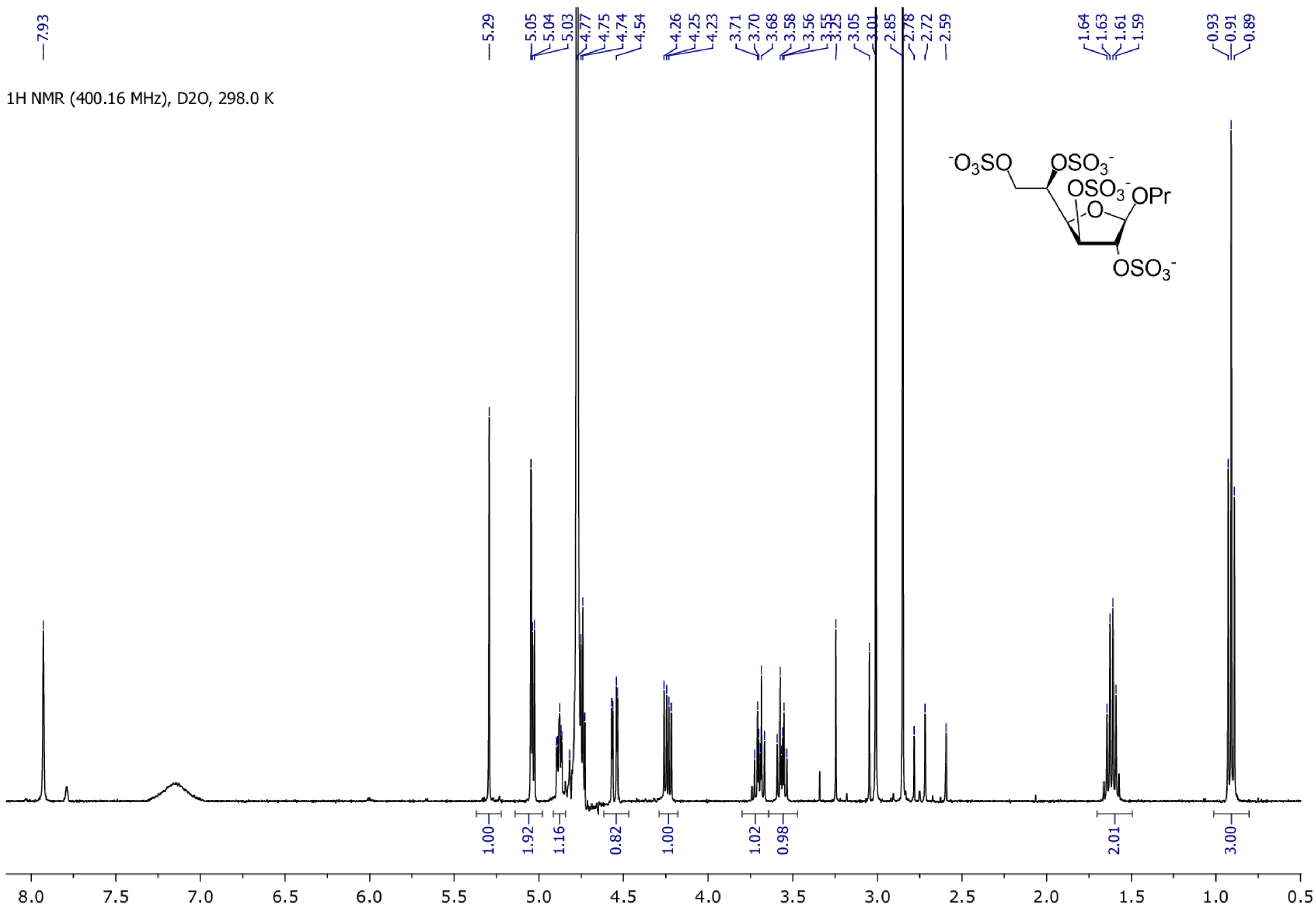

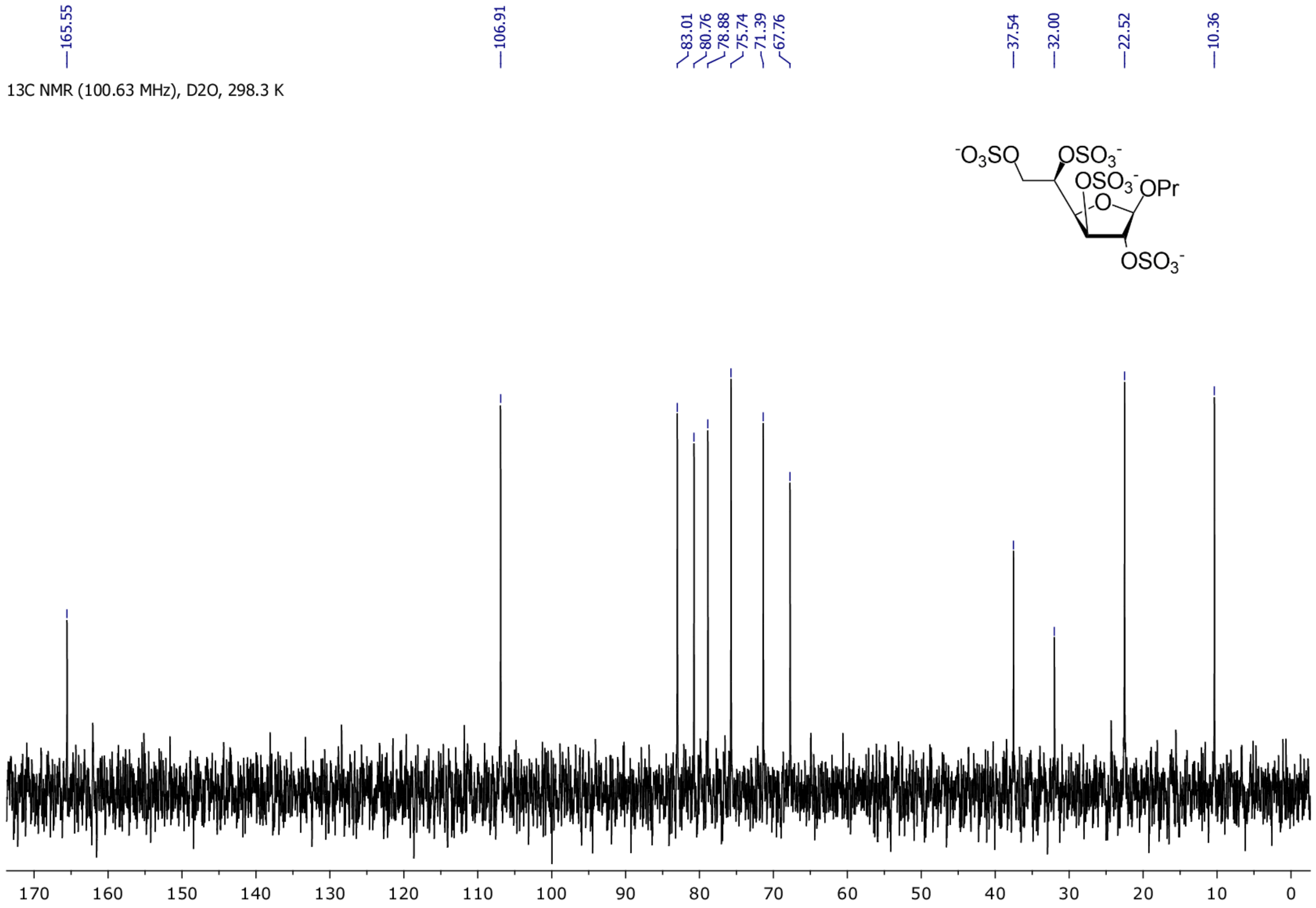

165.55
106.91
83.01
80.76
78.88
75.74
71.39
67.76
37.54
32.00
22.52
10.36
13C NMR (100.63 MHz), D2O, 298.3 K
-O3SO
OSO3-
OSO3-
OPr
O
OSO3-
170 160 150 140 130 120 110 100 90 80 70 60 50 40 30 20 10 0

Propyl β-D-galactofuranoside (3)

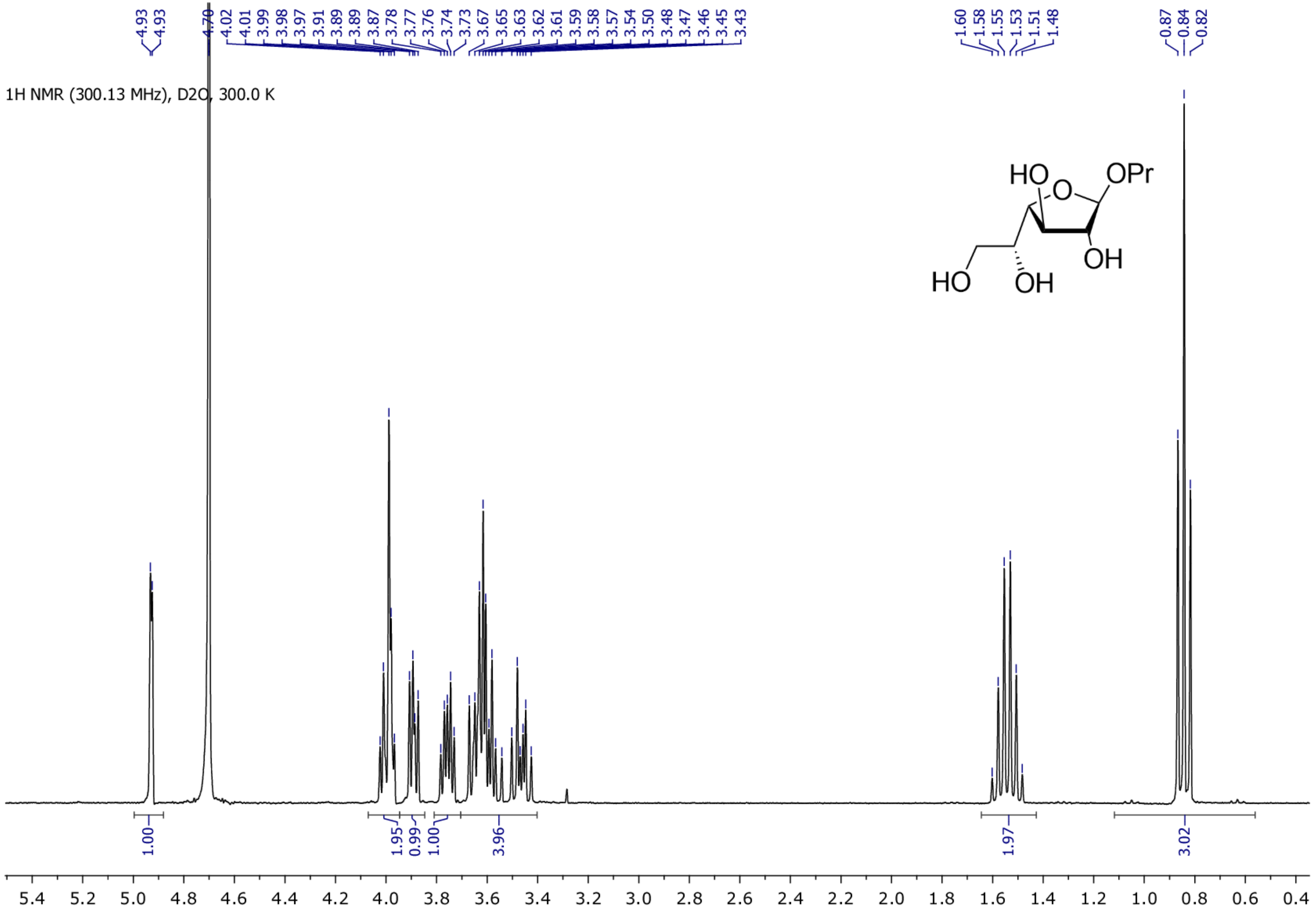

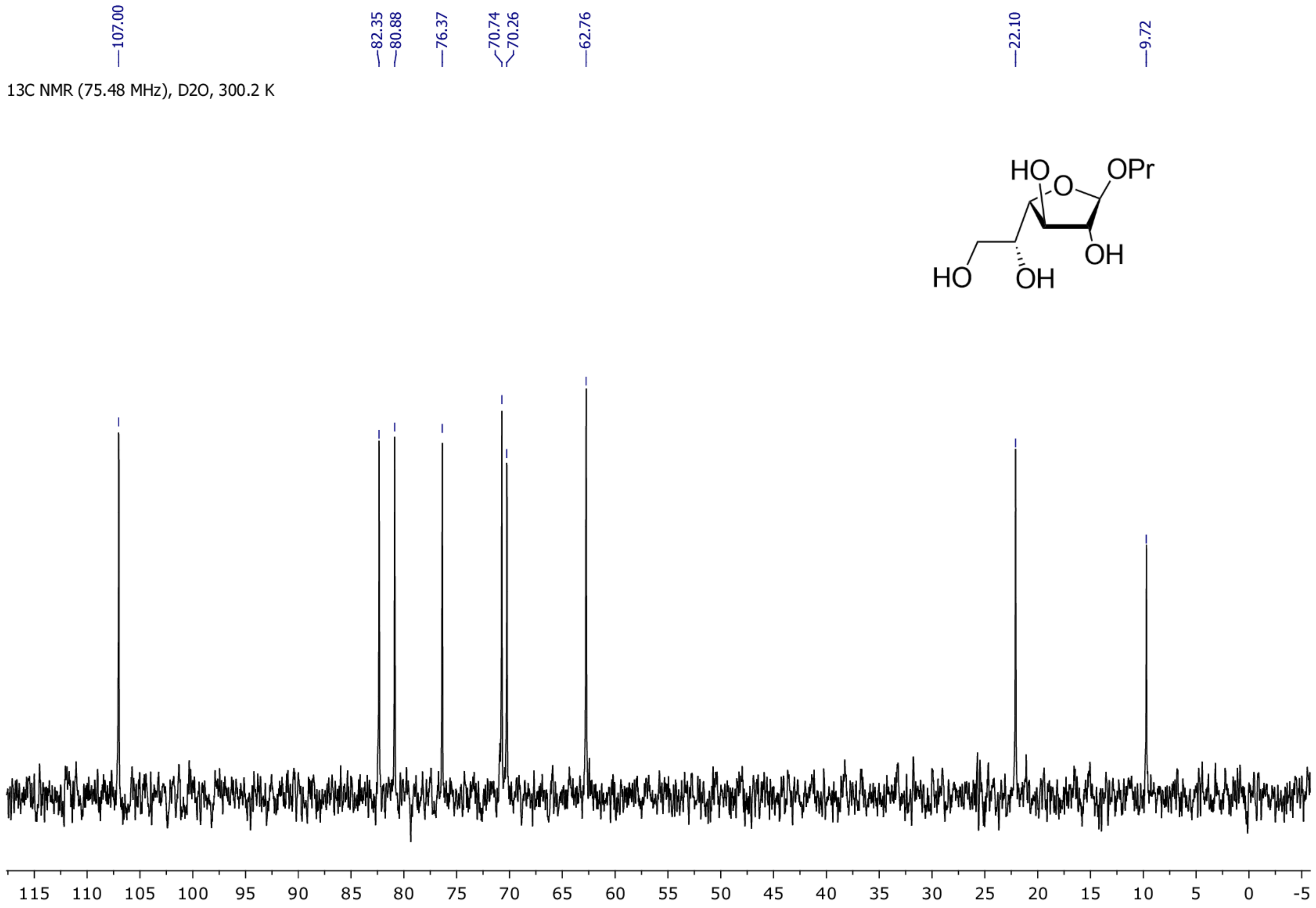
107.00
82.35
80.88
76.37
70.74
70.26
62.76
22.10
9.72
13C NMR (75.48 MHz), D2O, 300.2 K
HO
O
OPr
OH
HO
OH
115 110 105 100 95 90 85 80 75 70 65 60 55 50 45 40 35 30 25 20 15 10 5 0 -5

Per-O-sulfated propyl β-D-galactofuranoside (3s)

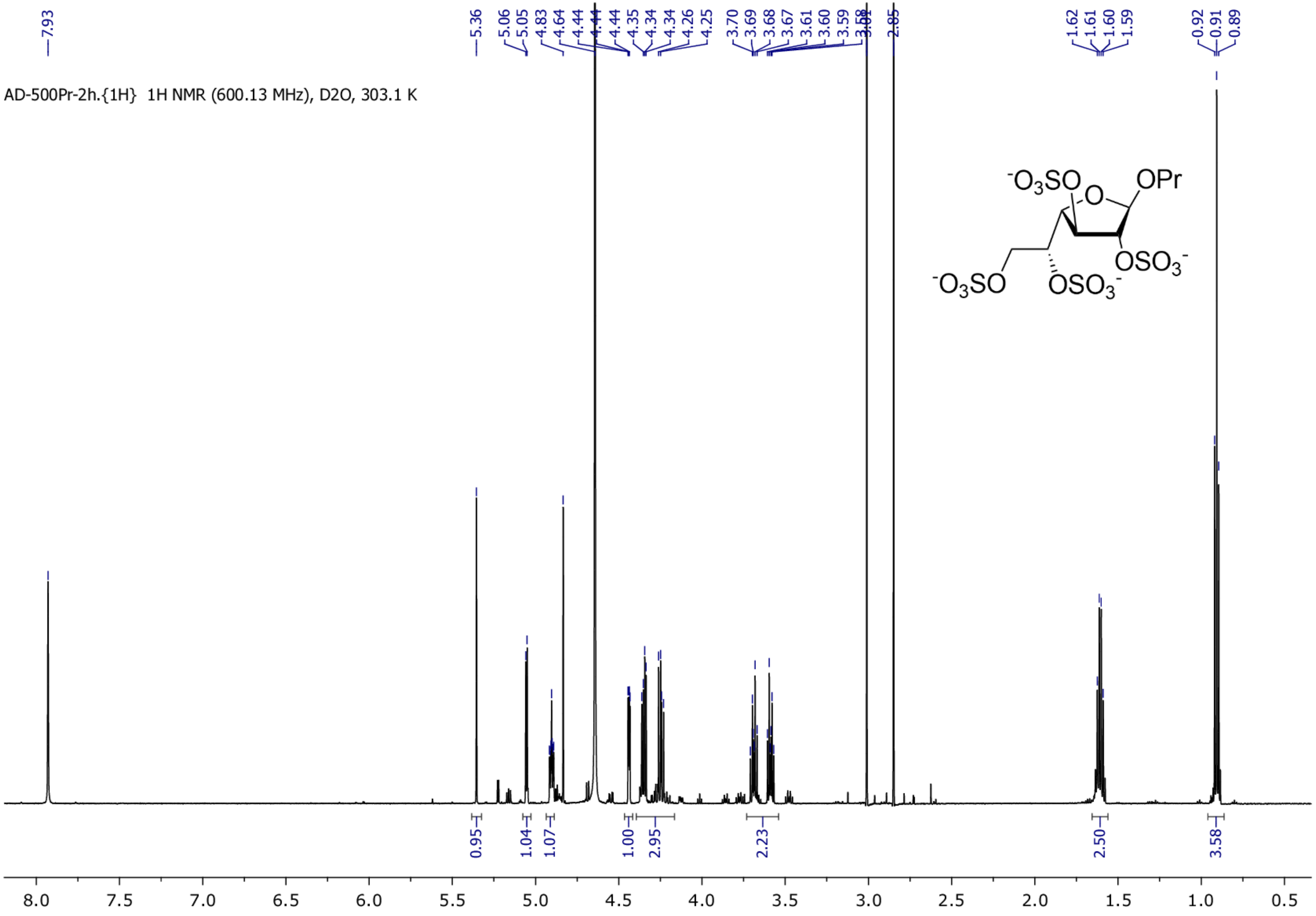

ad-500 13C NMR (100.63 MHz), D2O, 303.0 K

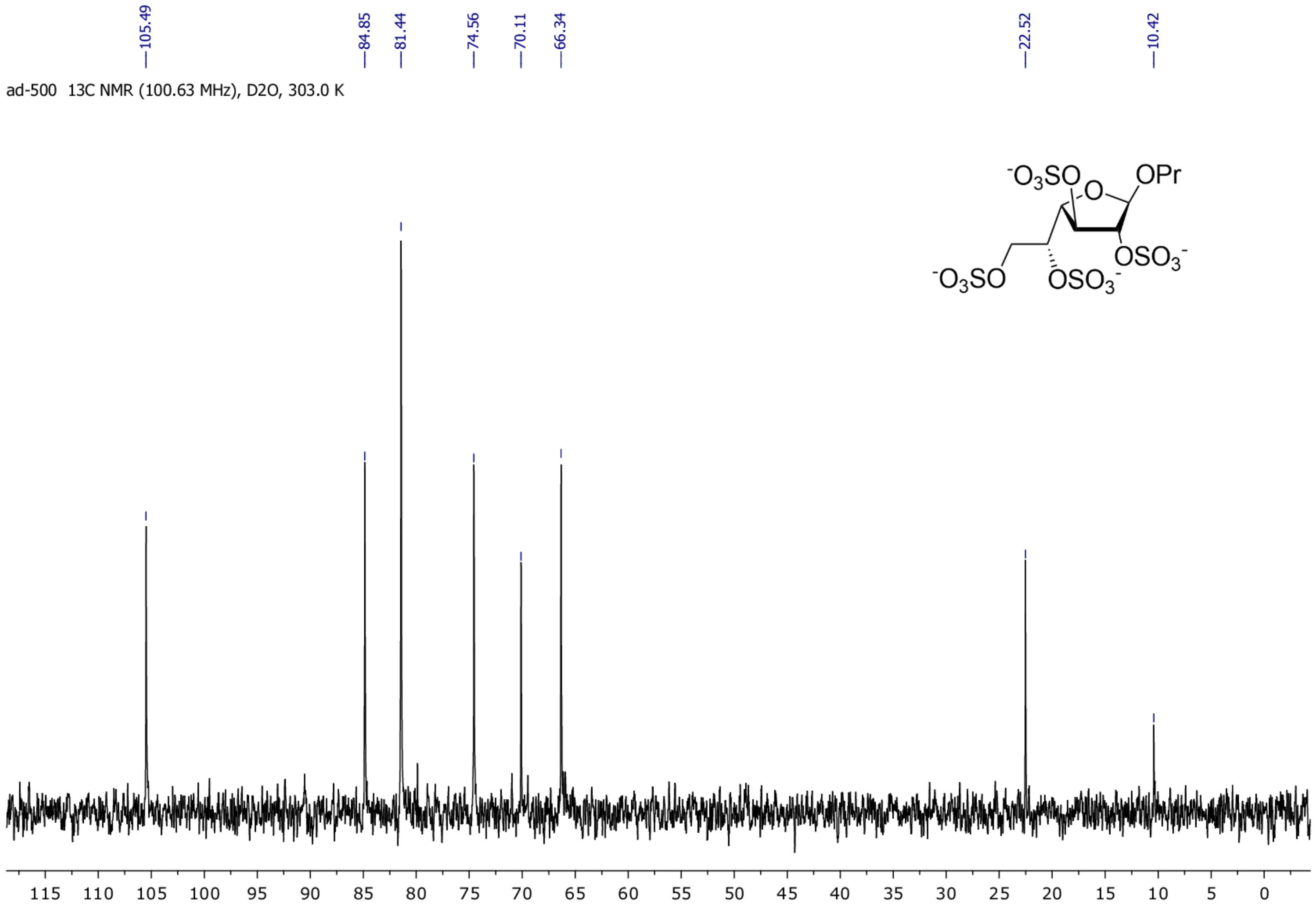

Table S1. Starting and final conformations with their energies for structure **1s**.

Starting conformer	Absolute energy, hartree	Energy relative to the lowest found conformation, kcal/mole	Final conformer
C1-endo_-60	-3287.431410778	7.2	C2-exo/C3-endo
C1-endo_+60	-3287.441204100	1.0	C2-exo/**C3-endo**
C1-exo_-60	-3287.434707545	5.1	C1-exo
C1-exo_+60	-3287.442860602	0.0	C1-exo
C2-endo_-60	-3287.434681089	5.1	C1-exo
C2-endo_+60	-3287.442815659	0.0	C1-exo
C2-exo_-60	-3287.441222255	1.0	C2-exo/**C3-endo**
C2-exo_+60	-3287.431381399	7.2	C2-exo/**C3-endo**
C3-endo_-60	-3287.431428682	7.2	C2-exo/**C3-endo**
C3-endo_+60	-3287.441426069	0.9	C2-exo/**C3-endo**
C3-exo_-60	-3287.432478471	6.5	C2-endo
C3-exo_+60	-3287.442816477	0.0	C1-exo
C4-endo_-60	-3287.432491076	6.5	C2-endo
C4-endo_+60	-3287.442776143	0.1	C1-exo
C4-exo_-60	-3287.431424486	7.2	C4-exo
C4-exo_+60	-3287.440999657	1.2	C4-exo
O4-endo_-60	-3287.434632933	5.2	C1-exo
O4-endo_+60	-3287.442822636	0.0	C1-exo
O4-exo_-60	-3287.432335961	6.6	C2-endo
O4-exo_+60	-3287.442825355	0.0	C1-exo

Table S2. Starting and final conformations with their energies for structure **1**.

Starting conformer	Absolute energy, hartree	Energy relative to the lowest found conformation, kcal/mole	Final conformer
C1-endo_-60	-800.697596820	4.1	C3-endo
C1-endo_+60	-800.700791611	2.1	C3-endo
C1-endo_180	-800.704085847	0.0	C3-endo
C1-exo_-60	-800.691467457	7.9	C1-exo
C1-exo_+60	-800.696711940	4.6	C1-exo
C1-exo_180	-800.699386724	2.9	C1-exo
C2-endo_-60	-800.692123482	7.5	C1-exo
C2-endo_+60	-800.696701951	4.6	C1-exo
C2-endo_180	-800.699403766	2.9	C1-exo
C2-exo_-60	-800.700506479	2.2	C3-endo
C2-exo_+60	-800.698252725	3.7	C3-endo
C2-exo_180	-800.703141067	0.6	C3-endo
C3-endo_-60	-800.700508978	2.2	C3-endo
C3-endo_+60	-800.698275031	3.6	C3-endo
C3-endo_180	-800.703163472	0.6	C3-endo
C3-exo_-60	-800.699947556	2.6	C2-endo
C3-exo_+60	-800.699741055	2.7	C1-exo
C3-exo_180	-800.703138229	0.6	C1-exo
C4-endo_-60	-800.699920198	2.6	C2-endo
C4-endo_+60	-800.699737988	2.7	C1-exo
C4-endo_180	-800.703163029	0.6	C1-exo
C4-exo_-60	-800.700502528	2.2	C3-endo
C4-exo_+60	-800.698257524	3.7	C3-endo
C4-exo_180	-800.703128644	0.6	C3-endo
O4-endo_-60	-800.703097903	0.6	C3-endo
O4-endo_+60	-800.699868677	2.6	C1-exo
O4-endo_180	-800.703133682	0.6	C1-exo
O4-exo_-60	-800.699183006	3.1	C2-endo
O4-exo_+60	-800.699838525	2.7	C1-exo
O4-exo_180	-800.703117696	0.6	C3-endo

Table S3. Starting and final conformations with their energies for structure **2s**.

Starting conformer	Absolute energy, hartree	Energy relative to the lowest found conformation, kcal/mole	Final conformer
C1-endo_-60	-3287.434834474	6.1	C2-exo/C3-endo
C1-endo_+60	-3287.444516980	0.1	**C2-exo**/C3-endo
C1-endo_180	-3287.437815713	4.3	C2-exo/C3-endo
C1-exo_-60	-3287.435433086	5.8	C4-endo/C3-exo
C1-exo_+60	-3287.433049873	7.3	C4-endo/C3-exo
C1-exo_180	-3287.429713775	9.4	C4-endo
C2-endo_-60	-3287.433182646	7.2	C4-endo/C3-exo
C2-endo_+60	-3287.439092049	3.5	C4-endo/C3-exo
C2-endo_180	-3287.429654903	9.4	C4-endo/C3-exo
C2-exo_-60	-3287.434875084	6.1	C2-exo/C3-endo
C2-exo_+60	-3287.444623266	0.0	C2-exo/C3-endo
C2-exo_180	-3287.437747850	4.3	C2-exo/C3-endo
C3-endo_-60	-3287.434859978	6.1	C2-exo/C3-endo
C3-endo_+60	-3287.444628043	0.0	C2-exo/C3-endo
C3-endo_180	-3287.437876406	4.2	C2-exo/C3-endo
C3-exo_-60	-3287.435391621	5.8	C4-endo/C3-exo
C3-exo_+60	-3287.439094383	3.5	C4-endo/C3-exo
C3-exo_180	-3287.430638553	8.8	C4-endo/C3-exo
C4-endo_-60	-3287.432996891	7.3	C4-endo/C3-exo
C4-endo_+60	-3287.439119103	3.5	C4-endo/C3-exo
C4-endo_180	-3287.430032129	9.2	C4-endo/C3-exo
C4-exo_-60	-3287.434828508	6.1	C2-exo/C3-endo
C4-exo_+60	-3287.444542571	0.1	C2-exo/C3-endo
C4-exo_180	-3287.437850169	4.3	C2-exo/C3-endo
O4-endo_-60	-3287.435453272	5.8	C4-endo/C3-exo
O4-endo_+60	-3287.433147687	7.2	C4-endo/C3-exo
O4-endo_180	-3287.438058189	4.1	C2-exo/C3-endo
O4-exo_-60	-3287.432986486	7.3	C4-endo/C3-exo
O4-exo_+60	-3287.439078389	3.5	C4-endo/C3-exo
O4-exo_180	-3287.437845892	4.3	C2-exo/C3-endo

Table S4. Starting and final conformations with their energies for structure **2**.

Starting conformer	Absolute energy, hartree	Energy relative to the lowest found conformation, kcal/mole	Final conformer
C1-endo_-60	-800.699803925	2.1	C2-exo
C1-endo_+60	-800.698775680	2.7	C2-exo
C1-endo_180	-800.702939349	0.1	C2-exo
C1-exo_-60	-800.696925869	3.9	C4-endo
C1-exo_+60	-800.691869147	7.0	C3-exo
C1-exo_180	-800.696230240	4.3	C2-endo
C2-endo_-60	-800.696377241	4.2	C3-exo
C2-endo_+60	-800.698152879	3.1	C2-exo
C2-endo_180	-800.702689639	0.2	O4-endo
C2-exo_-60	-800.699829914	2.0	C2-exo
C2-exo_+60	-800.698784061	2.7	C2-exo/C3-endo
C2-exo_180	-800.702907234	0.1	C2-exo
C3-endo_-60	-800.700689790	1.5	C2-exo
C3-endo_+60	-800.696355567	4.2	C2-exo
C3-endo_180	-800.703067297	0.0	C2-exo
C3-exo_-60	-800.696384827	4.2	C3-exo
C3-exo_+60	-800.698245183	3.0	C2-endo
C3-exo_180	-800.699952461	2.0	C3-exo
C4-endo_-60	-800.696357505	4.2	C3-exo
C4-endo_+60	-800.698244268	3.0	C2-endo
C4-endo_180	-800.700001707	1.9	C3-exo
C4-exo_-60	-800.699880111	2.0	C2-exo
C4-exo_+60	-800.698047689	3.2	C2-exo
C4-exo_180	-800.703087386	0.0	C2-exo
O4-endo_-60	-800.696928753	3.9	C4-endo/C3-exo
O4-endo_+60	-800.691877399	7.0	C3-exo
O4-endo_180	-800.696216494	4.3	C2-exo
O4-exo_-60	-800.696346302	4.2	C3-exo
O4-exo_+60	-800.698099176	3.1	C2-endo
O4-exo_180	-800.703098738	0.0	C2-exo

Table S5. Starting and final conformations with their energies for structure **3s**.

Starting conformer	Absolute energy, hartree	Energy relative to the lowest found conformation, kcal/mole	Final conformer
C1-endo_-60	-3287.462577288	2.2	C1-endo
C1-endo_+60	-3287.460456548	3.5	C1-endo
C1-endo_180	-3287.460664733	3.4	C1-endo
C1-exo_-60	-3287.462506805	2.2	C1-endo
C1-exo_+60	-3287.458028624	5.0	C1-exo
C1-exo_180	-3287.457152559	5.6	C3-endo
C2-endo_-60	-3287.462538150	2.2	C1-endo
C2-endo_+60	-3287.457976681	5.1	C1-exo
C2-endo_180	-3287.460416586	3.5	C1-endo
C2-exo_-60	-3287.456152731	6.2	C1-endo
C2-exo_+60	-3287.458663355	4.6	C1-endo
C2-exo_180	-3287.456739561	5.8	C1-endo
C3-endo_-60	-3287.462511036	2.2	C1-endo
C3-endo_+60	-3287.460471777	3.5	C1-endo
C3-endo_180	-3287.457115711	5.6	C3-endo
C3-exo_-60	-3287.462513615	2.2	C1-endo
C3-exo_+60	-3287.458651441	4.6	C1-exo
C3-exo_180	-3287.460655387	3.4	C1-endo
C4-endo_-60	-3287.458337015	4.8	O4-exo
C4-endo_+60	-3287.466057720	0.0	C1-endo
C4-endo_180	-3287.458441313	4.8	C1-endo
C4-exo_-60	-3287.456323521	6.1	C1-endo
C4-exo_+60	-3287.458079244	5.0	C1-exo
C4-exo_180	-3287.456717075	5.9	C1-endo/C2-exo
O4-endo_-60	-3287.456468019	6.0	C1-endo
O4-endo_+60	-3287.456965412	5.7	C1-exo
O4-endo_180	-3287.456989096	5.7	C3-endo
O4-exo_-60	-3287.452654291	8.4	C1-endo
O4-exo_+60	-3287.462089579	2.5	C1-endo
O4-exo_180	-3287.456760122	5.8	C1-endo

Table S6. Starting and final conformations with their energies for structure **3**.

Starting conformer	Absolute energy, hartree	Energy relative to the lowest found conformation, kcal/mole	Final conformer
C1-endo_-60	-800.702714639	1.8	C1-endo/O4-exo
C1-endo_+60	-800.703644764	1.2	O4-exo
C1-endo_180	-800.701461245	2.6	C1-endo/O4-exo
C1-exo_-60	-800.703938468	1.0	C3-exo
C1-exo_+60	-800.705606279	0.0	C3-exo
C1-exo_180	-800.703380269	1.4	C3-exo
C2-endo_-60	-800.703948654	1.0	C3-exo
C2-endo_+60	-800.705603785	0.0	C3-exo
C2-endo_180	-800.703246722	1.5	C3-exo
C2-exo_-60	-800.702716147	1.8	O4-exo
C2-exo_+60	-800.703637788	1.2	O4-exo
C2-exo_180	-800.701427620	2.6	O4-exo
C3-endo_-60	-800.702714091	1.8	O4-exo
C3-endo_+60	-800.703664558	1.2	O4-exo
C3-endo_180	-800.701457153	2.6	O4-exo
C3-exo_-60	-800.703947849	1.0	C3-exo
C3-exo_+60	-800.705592316	0.0	C3-exo
C3-exo_180	-800.703292763	1.5	C3-exo
C4-endo_-60	-800.698709341	4.3	C4-endo/C3-exo
C4-endo_+60	-800.703611569	1.3	C3-exo
C4-endo_180	-800.703927481	1.1	C3-exo
C4-exo_-60	-800.702612882	1.9	O4-exo
C4-exo_+60	-800.705590826	0.0	C3-exo
C4-exo_180	-800.701438249	2.6	O4-exo
O4-endo_-60	-800.703928620	1.1	C3-exo
O4-endo_+60	-800.705607657	0.0	C3-exo
O4-endo_180	-800.703345536	1.4	C3-exo
O4-exo_-60	-800.702732610	1.8	O4-exo
O4-exo_+60	-800.703718598	1.2	O4-exo
O4-exo_180	-800.701518924	2.6	O4-exo
